# An evolved ribosome-inactivating protein targets and kills human melanoma cells *in vitro *and *in vivo*

**DOI:** 10.1186/1476-4598-9-28

**Published:** 2010-02-03

**Authors:** Melissa C Cheung, Leigh Revers, Subodini Perampalam, Xin Wei, Reza Kiarash, David E Green, Aws Abdul-Wahid, Jean Gariépy

**Affiliations:** 1 Department of Pharmaceutical Sciences, University of Toronto, 144 College Street, Toronto, ON, M5S 3 M2, Canada; 2 Imaging Research, Sunnybrook Research Institute, 2075 Bayview Avenue, Toronto, ON, M4N 3 M5, Canada; 3 Department of Medical Biophysics, University of Toronto, 610 University Avenue, Toronto, ON, M5G 2 M9, Canada; 4 STTARR Innovation Centre, Radiation Medicine Program, Princess Margaret Hospital, University Health Network, 101 College Street, Toronto, ON, M5G 1L7, Canada; 5 Department of Radiation Physics, Radiation Medicine Program, Princess Margaret Hospital, University Health Network, 610 University Avenue, Toronto, ON, M5G 2 M9, Canada

## Abstract

**Background:**

Few treatment options exist for patients with metastatic melanoma, resulting in poor prognosis. One standard treatment, dacarbazine (DTIC), shows low response rates ranging from 15 to 25 percent with an 8-month median survival time. The development of targeted therapeutics with novel mechanisms of action may improve patient outcome. Ribosome-inactivating proteins (RIPs) such as Shiga-like Toxin 1 (SLT-1) represent powerful scaffolds for developing selective anticancer agents. Here we report the discovery and properties of a single chain ribosome-inactivating protein (scRIP) derived from the cytotoxic A subunit of SLT-1 (SLT-1A), harboring the 7-amino acid peptide insertion IYSNKLM (termed SLT-1A^IYSNKLM^) allowing the toxin variant to selectively target and kill human melanoma cells.

**Results:**

SLT-1A^IYSNKLM ^was able to kill 7 of 8 human melanoma cell lines. This scRIP binds to 518-A2 human melanoma cells with a dissociation constant of 18 nM, resulting in the blockage of protein synthesis and apoptosis in such cells. Biodistribution and imaging studies of radiolabeled SLT-1A^IYSNKLM ^administered intravenously into SCID mice bearing a human melanoma xenograft indicate that SLT-1A^IYSNKLM ^readily accumulates at the tumor site as opposed to non-target tissues. Furthermore, the co-administration of SLT-1A^IYSNKLM ^with DTIC resulted in tumor regression and greatly increased survival in this mouse xenograft model in comparison to DTIC or SLT-1A^IYSNKLM ^treatment alone (115 day median survival versus 46 and 47 days respectively; *P *values < 0.001). SLT-1A^IYSNKLM ^is stable in serum and its intravenous administration resulted in modest immune responses following repeated injections in CD1 mice.

**Conclusions:**

These results demonstrate that the evolution of a scRIP template can lead to the discovery of novel cancer cell-targeted compounds and in the case of SLT-1A^IYSNKLM ^can specifically kill human melanoma cells *in vitro *and *in vivo*.

## Background

**Figure 1:**
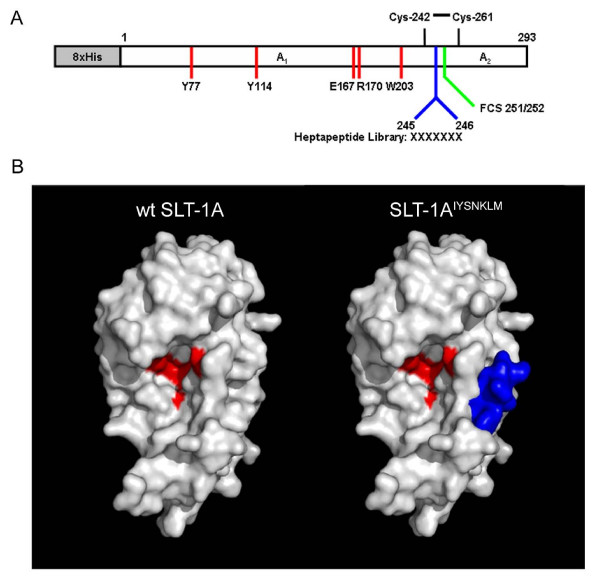
**The scRIP SLT-1 A subunit served as a scaffold for the design and construction of an embedded 7-amino acid peptide combinatorial SLT-1 A subunit library**. (A) Schematic diagram of the SLT-1 A subunit. Furin cleavage occurs within its protease-sensitive loop (C242 to C261) between residues 251 and 252 (FCS 251/252) of the A subunit [44]. A random 7-amino acid peptide library was inserted between residues 245 and 246, generating cytotoxic A subunit variants with a surface-displayed library insert. Catalytic residues are shown in red. (B) Modeled surface representations of the SLT-1 A subunit with and without a 7-residue long insertion. The models were derived from the crystal structure of Shiga Toxin [45]. The peptide insertion (blue) is solvent-exposed and positioned away from residues involved in catalysis (residues Y77, Y114, E167, R170, W203 colored in red) [20,46,47]. Models were rendered using PyMOL Software Version 1.0r1.

The incidence of melanoma has been rising in the United States for the past sixty years [1,2]. Despite prevention efforts, it remains the second leading cause of lost productive years among all cancers, and is responsible for more than 7,000 deaths annually [2]. Novel melanoma-targeted therapeutic agents are needed to improve prognosis, since traditional treatments such as dacarbazine (DTIC) and IL-2 only yield a 5% survival advantage of more than five years for patients with advanced melanoma [2]. Currently, targeted agents such as monoclonal antibodies and recombinant proteins account for more than a quarter of all cancer therapeutics that have been newly approved or are presently in clinical trials [3,4]. Although effective in delaying the progression of certain cancers, naked antibodies such as Herceptin and Avastin do not cure the disease [5-7]. This limitation has led to the use of tumor-targeted protein ligands in delivering potent therapeutic cargoes such as radionuclides, drugs, and protein toxins to tumor sites, bringing the overall concept of personalized medicine closer to reality [3,4]. In particular, immunotoxins and protein toxin conjugates have been the subject of clinical trials with a fusion construct of IL-2 to diphtheria toxin (Ontak) being approved by the FDA for the treatment of cutaneous T-cell lymphoma. Nevertheless, the use of toxin conjugates as successful cancer therapeutics remains limited [8-12]. To address some of the design challenges facing targeted protein toxins, we created a combinatorial protein library based on the cytotoxic domain of a protein toxin with a view to directly screen in cell-based assays for variants bearing new ligand specificities and able to selectively destroy cancer cells [13]. The approach makes use of the cytotoxic A subunit of a bacterial ribosome-inactivating protein (RIP), namely Shiga-like Toxin 1 (SLT-1), as a protein scaffold to design anticancer agents. Specifically, the SLT-1 A subunit encodes all functions necessary to route itself out of cellular organelles in order to reach and inactivate ribosomes present in the cytoplasm of eukaryotic cells. This event subsequently leads to apoptosis [14]. However, the SLT-1 A subunit lacks the ability to target cancer cells. In this report, we have inserted a random 7-amino acid peptide motif into the structure of the SLT-1A domain to create a combinatorial library of this protein template expressing toxic SLT-1A mutants harboring a putative peptide ligand that may specifically recognize, enter and kill cancer cells (Figure 1). Searching through such a library yielded a SLT-1A variant termed SLT-1A^IYSNKLM ^that selectively targets human melanoma cell lines. The mechanism of action and therapeutic activity of this single chain ribosome-inactivating protein (scRIP) variant closely parallel the predicted properties of a targeted ribosome-inactivating protein, suggesting that RIP A subunit libraries may represent a useful discovery tool for targeted protein-based therapeutics. We have termed this discovery approach RESCRIPT (Rapid Evolution and Selection of Cancer-specific Ribosome-Inactivating Protein Toxins) (see Additional File 1: Figure S1) which is based on the identification of targeted RIPs with no *a priori *knowledge of surface markers associated with cancer cells.

## Results

### Identification of SLT-1A^IYSNKLM^: a human melanoma-specific scRIP toxin variant

SLT-1 is a bacterial type II ribosome-inactivating protein produced by enteropathogenic *E. coli *strains such as O157:H7. Previously, the full-length SLT-1 (70 kDa; AB_5_) was used by our group as a scaffold to construct a combinatorial library. Random peptide elements were embedded within its receptor-binding B domain to alter receptor-binding specificity, while the wild type cytotoxic A subunit was retained to screen the library for SLT-1 variants able to selectively kill cancer cells [13]. However, the expression of protein variants from such a library was typically less than 1% when mutations were introduced in the receptor binding regions of the B subunit. We therefore describe here a novel combinatorial library designed using the smaller cytotoxic SLT-1 A subunit (31 kDa) as a scaffold (Additional File 2).

**Figure 2:**
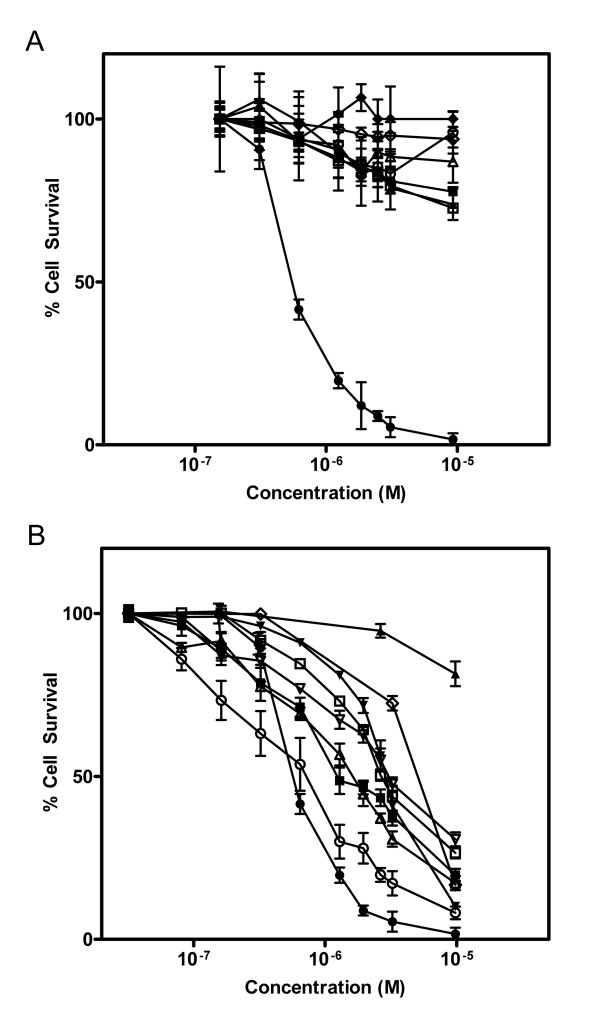
**Screening of the SLT-1 A subunit library yielded a toxin variant, SLT-1A^IYSNKLM^, displaying cytotoxicity towards human melanoma cell lines**. (A) Dose-response curves illustrating the specific cytotoxicity of SLT-1A^IYSNKLM ^(black diamonds, black triangles, black squares, black circles) compared to wt SLT-1A (white diamonds, white triangles, white squares, white circles) for the 518-A2 human melanoma cell line (black squares, white circles). The carcinoma cell lines CAMA-1 (black diamonds, white diamonds; human breast), HepG2 (black triangles, white triangles; human liver), and PC-3 (black squares, white squares; human prostate) are insensitive to SLT-1A^IYSNKLM^. (B) Dose-response curves illustrating the cytotoxicity profile of SLT-1A^IYSNKLM ^towards 8 different human melanoma cell lines: 518-A2 (black circles), A-375 (black inverted triangles), SK-Mel-28 (white circles), MeWo (black squares), A-2058 (white inverted triangles), MALME-3 M (white triangles), SK-Mel-2 (white squares), and C-32 (black triangles) as well as human melanocytes (white diamonds). Error bars represent s.e.m. for experiments performed in quadruplicate.

A combinatorial SLT-1A protein library was constructed by inserting a random 7-amino acid peptide element between residues 245 and 246 of the A subunit (Figure 1A and Additional File 2). The insertion of this heptapeptide did not affect the catalytic activity of SLT-1A (Additional File 3). This peptide element was also shown to be exposed on the surface of the A subunit (Additional File 3). Single bacterial colonies were picked from the library and led to the purification of 9,400 His-tagged scRIP variants that were tested for their ability to kill the wt SLT-1-resistant human melanoma cell line 518-A2 (see Additional File 1: Figure S1 and Additional File 2). The initial screens led to the recovery of 112 SLT-1A toxin variants displaying cytotoxic profiles towards cancer cell lines. These SLT-1A variants were re-screened against 518-A2 cells, as well as against a panel of twelve other cell lines (human unless otherwise indicated): PC-3 (prostate cancer), SKBR-3 (breast cancer), CAMA-1 (breast cancer), U87 (glioma), OVCAR-3 (ovarian carcinoma), SiHa (cervical cancer), PanC (pancreatic cancer), B16-F10 (mouse melanoma), Vero (monkey, normal kidney), HS-216 (normal fibroblast), H-MEC (normal mammary epithelial cells), and H-REC (normal kidney cells). This counter-selection step led to the identification of an A subunit toxin variant, named SLT-1A^IYSNKLM^, harboring the peptide sequence IYSNKLM, that selectively targets and kills 518-A2 cells in a dose-dependent manner [CD_50 _~300 nM] (Figure 2A). Furthermore, SLT-1A^IYSNKLM ^demonstrates broad specificity towards melanoma cell lines, killing 7 of 8 human melanoma cell lines tested (518-A2, A-2058, A-375, C-32, MALME-3 M, MeWo, SK-Mel-2, and SK-Mel-28), as well as human melanocytes to a lesser extent (Figure 2B).

### Catalytically active SLT-1A^IYSNKLM ^triggers apoptosis in human melanoma cells

**Figure 3:**
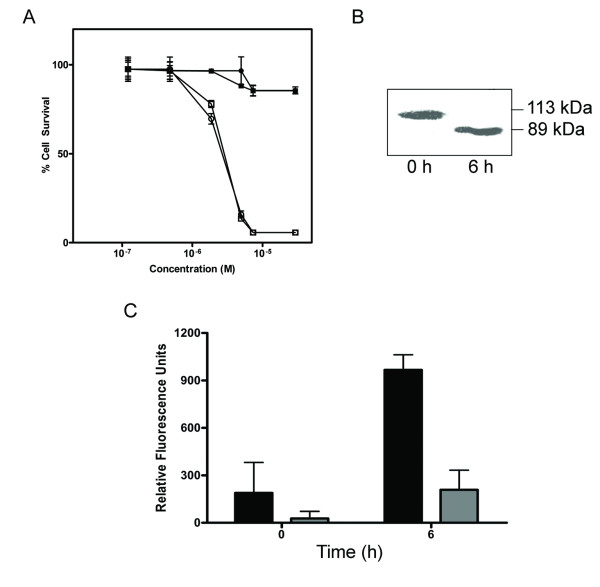
**The cellular activities of SLT-1A^IYSNKLM ^are linked to its catalytic activity**. (A) Dose-response curves indicating a comparable cytotoxicity for the purified, furin-cleaved A subunit of SLT-1A^IYSNKLM ^(A_1 _and A_2 _subunits remain linked by a disulfide bridge between C242 and C261; white squares) and the A_1 _subunit alone of SLT-1A^IYSNKLM ^(white circles) towards 518-A2 cells. The catalytically-inactive forms (Y77S) of these same molecules (black squares, black circles) were not cytotoxic towards 518-A2 human melanoma cells at concentrations of up to 10 μM. (B) Western blot analysis demonstrating the cleavage of PARP following treatment of 518-A2 cells with SLT-1A^IYSNKLM^. (C) Effect of exposing 518-A2 (black bars) and PC-3 cells (grey bars) to SLT-1A^IYSNKLM ^on the activation of caspase-3 as measured using a fluorescent peptide substrate for caspase-3. PC-3 cells are insensitive to SLT-1A^IYSNKLM^. Error bars represent s.e.m. for experiments performed in quadruplicate.

The cytotoxic A subunit of SLT-1 inactivates protein synthesis by depurinating an adenine residue (A_4324_) on 28S rRNA and inducing apoptosis [15-19]. To confirm that the toxicity for SLT-1A^IYSNKLM ^is in fact due to its catalytic activity, a key residue (Y77) was mutated to a serine within the A subunit of SLT-1, a substitution known to inactivate wt SLT-1 [20]. Cell survival assays indicated that 518-A2 cells are insensitive to the action of the catalytically inactive form of SLT-1A^IYSNKLM ^(Figure 3A). In addition, 518-A2 cells exposed to SLT-1A^IYSNKLM ^were shown to undergo apoptosis, as measured by the caspase-3 cleavage of a fluorescent peptide substrate for this enzyme or by the cleavage of Poly(ADP) ribose polymerase (PARP) (Figure 3B, C). In contrast, PC-3 cells were insensitive to the action of SLT-1A^IYSNKLM ^(Figure 2A) and their exposure to this scRIP resulted in only a modest level of caspase-3 activation (Figure 3C).

### SLT-1A^IYSNKLM ^binds to receptors on human melanoma 518-A2 cells

**Figure 4:**
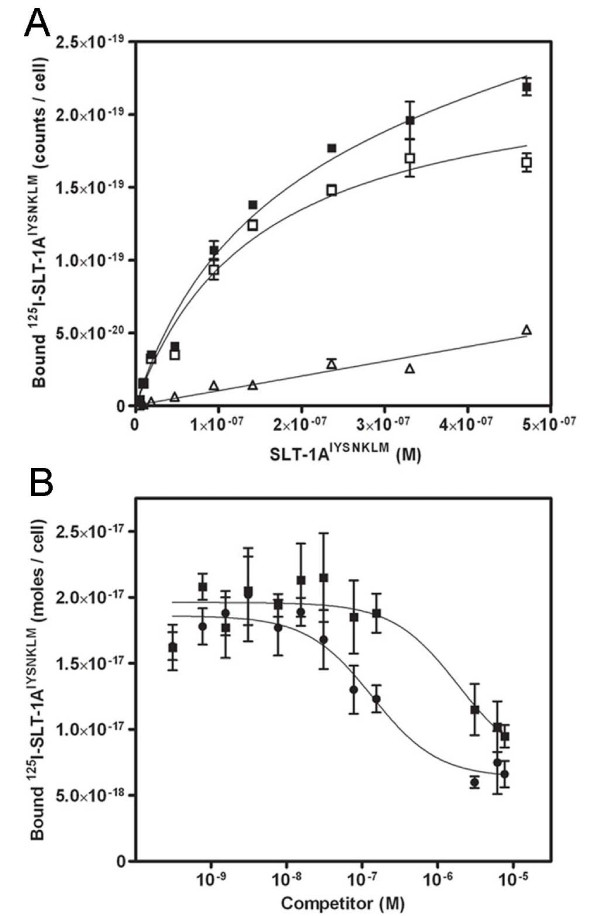
**Binding of ^125^I-SLT-1A^IYSNKLM ^to 518-A2 cells**. (A) Specific binding (white squares), total binding (black squares), and non-specific binding (white triangles) of ^125^I-SLT-1A^IYSNKLM ^to 518-A2 cells at 4°C. (B) Displacement curves in which 518-A2 cells were treated with 45 nM of ^125^I-SLT-1A^IYSNKLM ^in the presence of increasing concentrations of either unlabeled SLT-1A^IYSNKLM ^(black circles) or a synthetic 16-residue peptide containing the inserted 7-amino acid peptide ligand (HHH**IYSNKLM**ASRVAR) (black squares). Data points represent the s.e.m. of experiments performed in triplicate.

SLT-1A^IYSNKLM ^was radiolabeled with iodine-125 in order to further assess its properties towards 518-A2 cells *in vitro *and later *in vivo*. The dissociation constant of ^125^I-SLT-1A^IYSNKLM ^to receptors on human melanoma 518-A2 cells was derived at 4°C from a series of competition binding curves. Specifically, 518-A2 cells were incubated with ^125^I-SLT-1A^IYSNKLM ^ranging in concentration from 1.2 × 10^-9 ^M to 1.2 × 10^-6 ^M in the presence (non-specific binding) and absence (total binding) of a 100-fold excess of unlabeled SLT-1A^IYSNKLM^. The equilibrium dissociation constant (K^a^) for ^125^I-SLT-1A^IYSNKLM ^was calculated to be 1.4 (± 0.2) × 10^-7 ^M with 518-A2 human melanoma cells expressing 1.4 (± 0.1) × 10^5 ^binding sites for ^125^I-SLT-1A^IYSNKLM ^per cell (Figure 4A). The binding of ^125^I-SLT-1A^IYSNKLM ^to 518-A2 cells was also shown to be reversible. Displacement binding curves of ^125^I-SLT-1A^IYSNKLM ^bound to 518-A2 cells with unlabeled SLT-1A^IYSNKLM ^defined its inhibitory concentration (IC_50_) at 2.4 (± 0.3) × 10^-8 ^M (Figure 4B). Using this IC_50 _value, the dissociation constant (K_d_) of unlabeled SLT-1A^IYSNKLM ^was calculated to be 1.8 (± 0.3) × 10^-8 ^M (see Additional File 2: Supplementary Text). The ten-fold increase in binding affinity for the unlabeled form of SLT-1A^IYSNKLM ^in relation to its radiolabeled form (18 nM versus 140 nM) may reflect the fact that one of the 3 tyrosine residues available for iodination is located in the 7-amino acid insert region. In contrast, higher concentrations of the peptide, HHH**IYSNKLM**ASRVAR were needed to displace bound ^125^I-SLT-1A^IYSNKLM ^from cells (Figure 4B). This peptide corresponds to the sequence encompassing the 7-residue peptide binding domain of SLT-1A^IYSNKLM ^as well as flanking residues within the A subunit. The calculated K_d _for this peptide was 1.4 (± 2.5) × 10^-6 ^M. The peptide alone was thus more than 100-fold weaker as a ligand than within the context of SLT-1A^IYSNKLM ^suggesting that the tumor-targeting properties of SLT-1A^IYSNKLM ^are imparted by both the inserted peptide sequence and the SLT-1A scaffold.

### SLT-1A^IYSNKLM ^rapidly localizes at tumor sites *in vivo*

**Figure 5:**
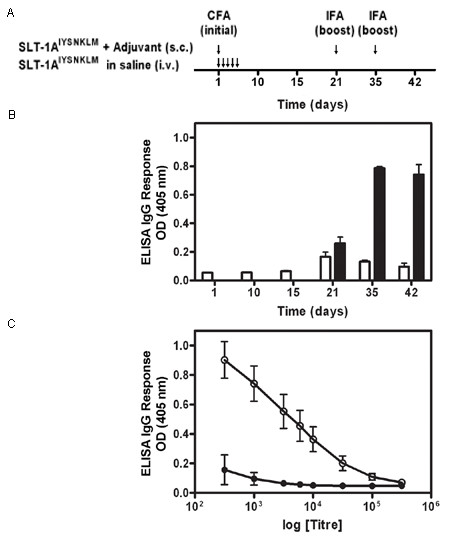
**SLT-1A^IYSNKLM ^generates modest IgG immune responses in CD1 mice**. (A) Injection schedule of SLT-1A^IYSNKLM ^with and without adjuvant. Treatments consisted of i.v. injections of SLT-1A^IYSNKLM ^prepared in saline into CD1 mice as a series of 5 consecutive (daily) tail vein injections or a single s.c. injection of SLT-1A^IYSNKLM ^in Complete Freund's adjuvant (CFA) followed by two s.c. boosts of the antigen in Incomplete Freund's adjuvant (IFA) at days 21 and 35. (B) Histogram illustrating the average IgG immune responses (1:1000 titer dilution) from 3 CD1 mice injected with SLT-1A^IYSNKLM ^in the presence (black bars) or absence of adjuvant (white bars) as measured by ELISA at 405 nm. (C) Representative IgG immune responses (average ELISA signals at 405 nm versus antisera titers; day 42) engendered for groups of three CD1 mice after injection with SLT-1A^IYSNKLM ^in the presence (white circles) or absence of adjuvant (black circles). Data represent the s.e.m. of experiments performed in triplicate.

In order to establish the *in vivo *properties of SLT-1A^IYSNKLM^, its stability was determined in human serum at 37°C. No substantial level of protein degradation was observed even after a 24 h exposure to 90% human serum (see Additional File 4: Figure S3). SLT-1A^IYSNKLM ^was also injected intravenously into groups of three CD1 mice as a series of 5 consecutive (250 μg/kg; daily) tail vein injections to assess antibody responses raised against this mutant A subunit (Figure 5A). Only modest IgG responses could be detected in three separate mice as compared with CD1 mice that had subcutaneously received the same antigen emulsified in Freund's complete adjuvant (Figure 5B). A representative set of IgG responses as a function of mouse serum dilutions at day 42 post-injection is also presented in Figure 5C. Finally, a tail vein injection of up to 4 mg/kg in mice did not result in any signs of toxicity (results not shown). Overall, these findings suggest that the repeated injections of SLT-1 A subunit variants such as SLT-1A^IYSNKLM ^elicit modest humoral responses in mice and compare well with responses observed in patients treated with other targeted toxin therapies [21,22].

**Figure 6:**
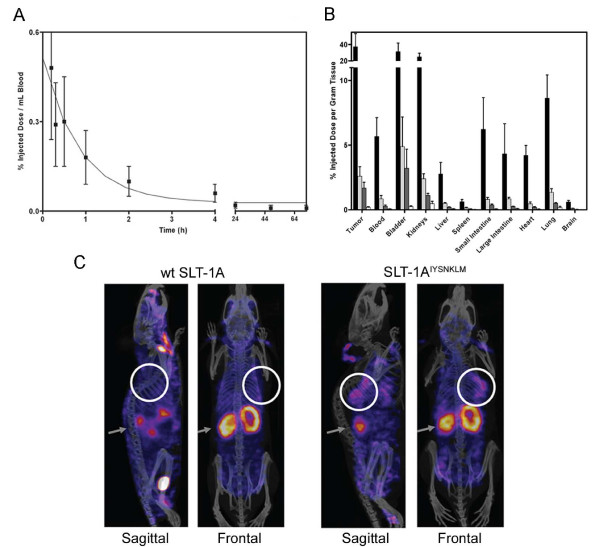
***In vivo *blood clearance rate and tumor-targeting of ^125^I-SLT-1A^IYSNKLM ^(A) Blood clearance of ^125^I-SLT-1A^IYSNKLM ^shown as percentage of injected dose per mL blood collected at various time points over a 72 h period post-i.v. injection**. The clearance rate (t_1/2_) of ^125^I-SLT-1A^IYSNKLM ^was shown to be ~11 min. (B) Biodistribution per collected gram of wet tissue of ^125^I-SLT-1A^IYSNKLM ^after 1 h (black bars), 6 h (light grey bars), 12 h (dark grey bars) and 24 h (white bars) post-i.v. administration. (C) Tumor localization of ^125^I-SLT-1A^IYSNKLM ^versus wt ^125^I-SLT-1A (negative control) as shown by nanoSPECT and CT imaging. The composite images show the tissue uptake of the radiolabeled proteins (as pink colored areas) as well as the location of tumor xenografts (white circles) and that of kidneys (grey arrows).

^125^I-SLT-1A^IYSNKLM ^was subsequently injected i.v. into SCID mice bearing 518-A2 tumor xenografts [23] to establish the pharmacokinetic parameters of this agent and its ability to localize into tumor cells. The scRIP was rapidly cleared through the kidneys with a circulating half-life (t_1/2_) of ~11 min, a value that is consistent with a protein of this size (Figure 6A) [24]. Importantly, the biodistribution profile showed marked tumor uptake and retention of ^125^I-SLT-1A^IYSNKLM^, reaching a maximum of ~37% injected dose per gram of tumor at 1 h post-i.v. injection (Figure 6B). Tissue analyses also demonstrated the selectivity of ^125^I-SLT-1A^IYSNKLM ^in that tumor localization increases as the molecule is eliminated more slowly from the tumor than from blood and other tissues. Specifically, the tumor-to-blood ratios of ^125^I-SLT-1A^IYSNKLM ^at 1 h, 6 h, 12 h, and 24 h post-injection are 6.6, 3.1, 5.9 and 3.0 respectively.

To visualize the tumor-localization of SLT-1A^IYSNKLM^, a group of 518-A2 xenograft-bearing SCID mice received a single i.v. injection of either ^125^I-SLT-1A^IYSNKLM ^(900 μCi) or wt ^125^I-SLT-1A (900 μCi; negative control). The distribution of radiolabeled scRIPs was recorded 45 min post-injection with a nanoSPECT/CT scanner. The resulting CT MIP (Maximum Intensity Projection) images were then overlaid with the SPECT image slice that transversed the central plane of the tumor (Figure 6C). These images highlight the rapid localization and specificity of ^125^I-SLT-1A^IYSNKLM ^into 518-A2 cell xenografts as compared to the control, wt ^125^I-SLT-1A, which shows no tumor localization.

### SLT-1A^IYSNKLM ^causes tumor regression and increased survival *in vivo*

**Figure 7:**
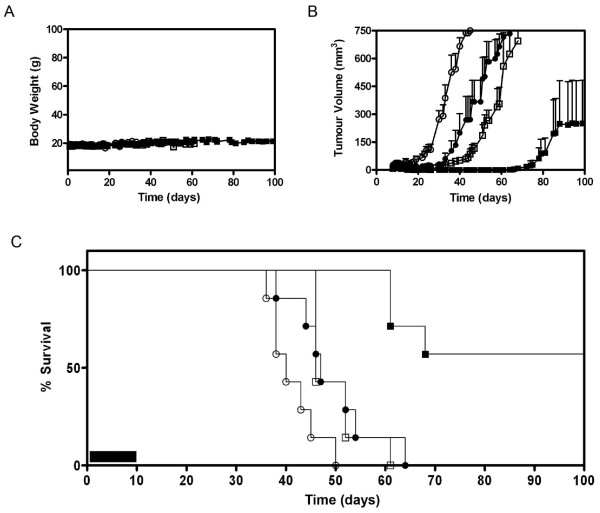
***In vivo *results following treatment regimens in SCID mice harboring established 518-A2 tumor xenografts**. The regimens included 10-day, daily injections of a saline control (i.v) (white circles) or SLT-1A^IYSNKLM ^(i.v. 0.5 mg/kg) (black circles), or a 5-day course of DTIC (i.p. 8 mg/kg) (white squares), or the combination of both SLT-1A^IYSNKLM ^(i.v. 0.5 mg/kg) with DTIC (i.p. 8 mg/kg) regimens (black squares), (n = 7). (A) Mice showed no change in body weight related to treatment regimens over time. (B) Measured tumor volumes of mice demonstrate a significant synergistic effect of combining SLT-1A^IYSNKLM ^and DTIC treatments when compared to either treatment alone or to the saline control (*P *< 0.0001). (C) Kaplan-Meier plot comparing animal survival as a function of treatment regimens (ten-day regimen; black bar).

The final component in determining the usefulness of SLT-1A^IYSNKLM ^was to assess the value of this scRIP in improving the survival time of 518-A2 xenograft-bearing SCID mice [23]. When tumors reached a volume of ~30 mm^3^, animals received daily i.v. injections for ten days of either saline, SLT-1A^IYSNKLM^, or a 5-day i.p. regimen of DTIC (a standard-of-care chemotherapeutic agent for patients with advanced melanoma) or a combination treatment involving both SLT-1A^IYSNKLM ^and DTIC. Animal body weight, tumor volume and survival were subsequently monitored over a period of 230 days (Figure 7). Body weight remained constant for all treatment groups with no statistically significant amount of weight loss being recorded during and after a treatment (Figure 7A). Tumor-bearing mice showed complete tumor regression after a 10-day treatment with SLT-1A^IYSNKLM ^leading to a statistically significant (*P *< 0.05) increase in median survival from 40 days for the saline-treated group to 47 days. This gain in survival was comparable to the 46-day median survival observed for tumor-bearing mice treated with DTIC alone (Figure 7B and 7C; *P *= 0.86). The tumors eventually re-appeared following the completion of the 10-day regimen with SLT-1A^IYSNKLM ^(Figure 7C). In contrast, mice treated with the combination of SLT-1A^IYSNKLM ^and DTIC demonstrated an impressive median survival of 115 days, shown to be statistically significant as assessed using the Mantel-Cox test (*P *< 0.0001). This combination therapy resulted in some mice demonstrating no tumour recurrence, suggesting that it is the result of marked additive effects in combining therapies.

## Discussion

The incidence of melanoma has been rapidly rising worldwide with no effective treatment in sight for patients with metastatic forms of this disease [1]. Despite decades of evaluating new therapeutic modalities for advanced melanoma, patients are mainly being treated with agents that display low response rates, i.e. DTIC and IL-2 [2]. In view of the urgent need for new therapeutic strategies, we designed and screened a combinatorial library based on the scaffold of a single chain ribosome-inactivating protein (scRIP), namely the cytotoxic A subunit of SLT-1, to identify SLT-1 A variants that target and kill human melanoma cells. In this study, we demonstrated that the screening of this combinatorial SLT-1A protein library led to the identification of a toxin variant termed SLT-1A^IYSNKLM ^that selectively binds to human melanoma cells, is internalized by them and results in their apoptotic death.

Immunotoxins derived from the fusion of the cytotoxic domain of *Pseudomonas *exotoxin A (PE) to a tumor-targeted Fv fragment, have been shown to kill cells using a combination of cytotoxic and cytostatic mechanisms of action, namely the induction of apoptosis and cell death (cytotoxic mechanism) due to the inhibition of protein synthesis (cytostatic effect) [25,26]. Wild type SLT-1 utilizes both mechanisms. Firstly, the SLT-1 A subunit undergoes retrograde transport to the endoplasmic reticulum, where it then retrotranslocates to the cytoplasm and halts protein synthesis via its catalytic activity by cleaving 28S rRNA [27]. The blockage of protein synthesis subsequently triggers apoptosis through the activation of caspases 8, 9, and 3 leading to the display of morphological characteristics such as membrane blebbing, DNA fragmentation, chromatin condensation and cell shrinkage [14,17]. As presented in Figure 3, only the catalytically active SLT-1A^IYSNKLM ^kills cells, activates caspases-3 and cleaves PARP, as observed for ricin, another common RIP family member [28]. Thus, one key advantage of SLT-1A as a combinatorial library template is that it yields small scRIP agents with short new ligand binding domains without altering the inherent intracellular localization and cytotoxic properties of the A subunit. This mechanism of action is distinct from most commonly prescribed anticancer agents in the clinic, suggesting that the cytotoxic A subunit of SLT-1 could complement the action of existing drugs in combination therapy.

The melanoma-specific scRIP SLT-1A^IYSNKLM ^was directly derived using RESCRIPT (Additional File 1: Figure S1) from a cell-based screen and its therapeutic potential confirmed *in vivo *without the need to re-engineer parts of its structure. This discovery and optimization approach differs from preexisting immunotoxin design strategies. Specifically, the traditional two-step assembly of immunotoxins and related conjugates has been based on an *a priori *selection of a known tumor marker. However, the fusion or chemical coupling of a protein ligand (typically > 25 kDa) to a toxin domain often generates large protein constructs displaying altered functions in terms of cell targeting, cellular processing and cytotoxic functions [9,22,29]. Secondly, RESCRIPT, as a discovery tool, is distinct from phage display approaches which are typically used to identify peptide and protein ligands and are not presently compatible with cell killing assays. RESCRIPT thus limits the need for post-discovery, protein engineering steps required to ensure that functions such as cell targeting, cellular routing and toxicity are retained by the resulting conjugates.

Mechanistically, SLT-1A^IYSNKLM ^behaves as a catalytically active RIP causing apoptosis in 518-A2 melanoma cells (Figure 3). SLT-1A^IYSNKLM ^was shown to bind to a surface receptor on 518-A2 cells with a K^d ^of 18 nM. These cells express about 140,000 copies of the receptor. More importantly, the specific binding of this A subunit variant to receptors on melanoma cells was rapid, saturable and reversible (Figure 4). Preliminary biochemical studies aimed at determining the nature of the surface determinant recognized by SLT-1A^IYSNKLM ^on 518-A2 melanoma cells, suggests that the receptor may not be a protein. Specifically, pre-treating 518-A2 cells extensively with trypsin yielded an identical SLT-1A^IYSNKLM ^cell cytotoxicity profile to that of untreated 518-A2 cells. Furthermore, preliminary cell surface radioiodination followed by crosslinking/pull-down experiments with His-tagged SLT-1A^IYSNKLM ^and wt SLT-1A did not reveal any unique radiolabeled membrane species that may act as putative SLT-1A^IYSNKLM ^receptors on SDS-PAGE. Both approaches have limitations in terms of specificity and detection sensitivity. Biochemical and proteomic approaches are on-going to define this melanoma-specific receptor. Studies are also being conducted to determine whether the SLT-1A^IYSNKLM ^receptor may be a cell-surface melanoma marker that has already been described in the literature.

Although several melanoma biomarkers have previously been reported, only a few are expressed on the cell-surface and therefore would qualify as possible SLT-1A^IYSNKLM ^receptor candidates [30]. These surface markers include HMW-MAA (high molecular weight melanoma associated antigen; melanoma chondroitin sulfate proteoglycan; MCSP), S100B (originally known as S100), CD44, CXCR4 (CXC chemokine receptor) and CEACAM1 (carcinoembryonic-antigen-related cell adhesion molecule 1) [31-37]. However, these known markers are present on normal tissues as well as other cancer types, highlighting challenges in designing melanoma-specific therapies through the traditional method of conjugating a ligand to a cell-killing component [30,33,36,38,39]. Nevertheless, antibodies raised against some of these biomarkers, such as anti-Id mAb MK2-23 for HMW-MAA, have shown promise in increasing patient survival. To date, there is still a lack of clinically successful melanoma-specific markers, and none for the detection of primary tumors for high risk patients [30,40,41].

The pharmacological properties of SLT-1A^IYSNKLM ^were also investigated in a mouse model and suggest that the molecule is cleared with the expected half-life profile of antibody fragments with a mass of ~30 kDa. SLT-1A^IYSNKLM ^is also stable in human serum, displays modest immunogenicity, and accumulates readily in tumor xenografts of 518-A2 cells implanted in SCID mice (Figure 6, Figure S4 and Figure 5 and 7). This scRIP also retains its cytotoxic activity both *in vitro *(Figure 2 and 3) and *in vivo *as shown by the level of tumor regression observed upon treating tumor-bearing mice (Figure 7). These remarkable properties suggest that SLT-1A^IYSNKLM ^be pursued as a possible treatment modality for melanoma patients displaying metastatic forms of this disease.

## Conclusions

We have developed and screened a combinatorial single chain, ribosome-inactivating protein (scRIP) library with a view to use the intrinsic cytotoxicity of all members of the library (built-in search engine) as a tool to identify melanoma-specific scRIP variants in the context of cell viability assays. Library searches led to the discovery of SLT-1A^IYSNKLM^, a novel scRIP variant of the cytotoxic A subunit of Shiga-like Toxin 1 that is able to target and specifically kill human melanoma cells. SLT-1A^IYSNKLM ^represents a candidate agent for treating patients with metastatic melanoma.

## Materials and methods

### Antibodies, Cell Lines and Bacterial Strains

The 518-A2 human melanoma cell line was kindly provided by Dr. Burkhard Jansen [23] and maintained in Dulbecco's Modified Eagle Medium containing 5% FBS, 100 U/mL penicillin and 0.1 mg/mL streptomycin. All other cell lines were purchased from ATCC and grown according to company guidelines.

### Library Design

Recombinant SLT-1A variants were generated using a modified version of the method previously described by our group [13]. The initial library was designed to validate the variability of a heptapeptide insert as well as its effect on SLT-1 A subunit cytotoxicity (see Additional Files 2 and 3). The final heptapeptide library screened was genetically inserted into the A subunit of His-tagged SLT-1 between residues 245 and 246 by PCR. Specifically, the randomized insert was introduced by amplifying a fragment of the SLT-1 gene using primers A (CTG AAG CTT TAC GTT TTC GGC) and C (GAT GCC ATT CTG GCA ACT CGC GAT GCS NNS NNS NNS NNS NNS NNS NNS TGA TGA TGA CAA TTC AGT ATT AAT GC). This product was ligated to the remaining fragment, which had been amplified using primers B (GAG ACT GCA GAT TCC ATC TGT TGG) and D (GCA TCG CGA GTT GCC AGA ATG GCA), by PCR using primers A and B. After digestion with HindIII and PstI, the resulting library cassette was ligated into pECHE9A (pUC19-based plasmid that constitutively expresses SLT-1 constructs in bacteria), digested with NsiI to linearize wild-type material, transformed and expressed in JM101 *E. coli *(Promega).

### Expression and Purification of SLT-1A Subunit Library Variants

Individual library colonies were picked from carbenicillin-selective LB-agar plates and grown overnight at 37°C in 1 mL of LB-broth in 96-well culture blocks. Bacterial pellets were lysed in a single freeze-thaw cycle with BugBuster Protein Extraction Reagent (Novagen) supplemented with 2.5 U/g pellet of Benzonase nuclease (EMD Biosciences). Cleared lysates were transferred to 96-well plates containing 2 μL of Ni-NTA magnetic agarose beads (Promega) and incubated for 30 minutes at room temperature. The beads were washed with Buffer A (50 mM phosphate buffer, pH 8, 300 mM NaCl, and 10 mM imidazole), and eluted with Buffer C (same as Buffer A but with 250 mM imidazole). The generation of a catalytically-inactive form of SLT-1A^IYSNKLM ^is described in Additional File 2.

### Large-Scale Protein Purification

JM101 *E. coli *(Promega) transformants of SLT-1A^IYSNKLM ^(or the wt SLT-1A control) were grown in LB-broth containing 100 μg/mL ampicillin with shaking (225 rpm) at 37°C overnight. Each gram of cell pellet was resuspended in 5 mL of BugBuster Reagent (Novagen), 2.5 mL Buffer A (50 mM phosphate buffer, pH 8, 300 mM NaCl, and 10 mM imidazole), and 2.5 U Benzonase (2.5 kU; Novagen), and an EDTA-free protease inhibitor tablet (Roche). After stirring at room temperature for 1 h followed by centrifugation at 8,000 × g for 30 min, the cleared lysate was then loaded onto Ni-NTA agarose (Sigma-Aldrich) pre-equilibrated with Buffer A. The noncovalently-associated B subunit was removed from the bound His-tagged A subunit by treating the column with 6 M guanidine-HCl in Buffer A. The retained A subunits were renatured on the nickel resin by washing with Buffer A containing decreasing concentrations of guanidine-HCl [4.8 M, 3.6 M, 2.4 M, and 1.2 M respectively]. After an additional wash with Buffer B (50 mM phosphate buffer, 300 mM NaCl, 20 mM imidazole, pH 8), the purified A subunit was eluted with Buffer C, concentrated and dialyzed against saline using an Amicon ultrafiltration unit (Millipore; 10 kDa MWCO). Proteolytic cleavage was achieved by incubating the purified A subunit with recombinant furin protease (New England Biolabs) in 100 mM MES buffer (pH 6.0), 5 mM CaCl_2 _and 1 mM β-mercaptoethanol. Typically, 1 mg of SLT-1 A subunit (or SLT-1A^IYSNKLM^) was cleaved with 10 U of furin for 48 h at 30°C. The bacterial strains producing either wt SLT-1 or AB_5 _SLT-1A^IYSNKLM ^typically led to the recovery of 7.5 (+/- 2.5) mg of His-tagged purified toxins per liter of bacterial medium. On average, 2 mg of purified, furin-digested SLT-1A^IYSNKLM ^and 1.5 mg of purified, furin-digested wt SLT-1A were obtained per liter of bacterial culture. Samples were characterized by SDS-PAGE, and stained with Coomassie Blue. The purity of the final products was assessed by densitometry to be > 98% pure (See Additional File 5; Figure S4).

### Cell Viability and Apoptosis Measurements

Cells were exposed to escalating concentrations of purified SLT-1A^IYSNKLM ^or purified scRIP library variants for 1 h at 37°C followed by a 48 h incubation period in fresh medium. The viability of adherent cells was subsequently determined using the sulforhodamine B assay [42]. Apoptosis as measured by the activation of caspase 3 was determined after treating cells with 1 μM SLT-1A^IYSNKLM ^for 6 h. Caspase 3 proteolytic activity was assessed via the cleavage of the peptide substrate Ac-DEVD-AFC (SensoLyte Homogeneous AFC caspase-3/7 assay kit; ANASPEC) and of PARP [43] by immunoblotting with a rabbit polyclonal antibody to human ARP (Cell Signaling Technology).

### ^125^I Radiolabeling and Radioligand Binding Assays

SLT-1A^IYSNKLM ^and SLT-1A subunits were radioiodinated with carrier-free Na^125^I (37 MBq/10 uL; MP Bio) and Iodogen (Pierce). Radiolabeled A subunits were purified from unincorporated ^125^I by gel permeation (D-Salt Polyacrylamide, Pierce) and the specific activity determined and counted in a Wallac Wizard 3" 1480 instrument (PerkinElmer). The specific activity obtained was 2.1 × 10^17 ^cpm/mol and 7.1 × 10^16 ^cpm/mol for the SLT-1A^IYSNKLM ^and wt SLT-1A subunits respectively. Data was plotted and analyzed using GraphPad Prism 5.0 (GraphPad Software, Inc). Details regarding radioligand binding assays are available in Additional File 2.

### Pharmacokinetic, Biodistribution, Immunogenicity and Imaging Studies

Human melanoma 518-A2 cells (4 × 10^6 ^cells suspended in 0.2 mL culture medium) were implanted subcutaneously into five-week-old female SCID mice [23], resulting in tumor xenografts (volume of ~30 mm^3^) within 14 days. For pharmacokinetic analyses, tumor-bearing mice received a single i.v. injection of either ^125^I-SLT-1A^IYSNKLM ^or wt ^125^I-SLT-1A (14 μg, 150 μl dose in USP grade saline; 100 μCi). Blood samples were collected via the saphenous vein and counted in a gamma counter to determine blood clearance rates (4 mice per group). For biodistribution studies, a separate group of tumor-bearing SCID mice was anesthetized using 5% isoflurane gas and blood samples collected via cardiac puncture. Mice were sacrificed and tissues collected, weighed and counted in a gamma counter (n = 4). Results were reported as percent injected dose per gram of wet tissue weight.

Immunogenicity studies were performed with CD1 mice (3 mice per time point) given a series of 5 consecutive (250 μg/kg; 100 μL daily) i.v. injections of SLT-1A^IYSNKLM ^prepared in saline to mimic the therapeutic treatment procedure. As a positive control, a single dose (250 μg/kg; 100 μL) of this A chain emulsified (1:1) in Complete Freund's adjuvant (CFA) was injected subcutaneously into CD1 mice (control group). The control animals also received s.c. injections of the same antigen emulsified (1:1) in Incomplete Freund's adjuvant (IFA; 250 μg/kg; 100 μL dose) at day 21 and 35. To assess IgG immune responses raised against SLT-1A^IYSNKLM^, blood samples were collected at various time intervals post-injection (day 1, 10, 15, 21, 35, 42 for SLT-1A^IYSNKLM^; day 21, 35, 42 for the antigen with adjuvant). IgG responses were titrated for each mouse by serially diluting serum samples and measuring IgG levels by ELISA in 96-well plates pre-coated with 100 ng of SLT-1A^IYSNKLM^. Serum-treated wells (in triplicate) were subsequently exposed to an HRP-conjugated goat-anti-mouse IgG (1:1000) and ELISA signals recorded with a plate reader at 405 nm using the substrate ABTS (2,2'-Azinobis [3-ethylbenzothiazoline-6-sulfonic acid]-diammonium salt).

Imaging experiments were carried out at the UHN STTARR facility using a dual-modality NanoSPECT/CT (Bioscan) and are further described under Additional File 2 (2 mice). All animal protocols were reviewed and approved by the Animal Care Committee at the University Health Network.

### Tumor Regression Studies

The effects of SLT-1A^IYSNKLM ^and DTIC treatment on the survival of 518-A2 tumor-bearing SCID mice [23] were assessed when tumor xenografts reached a volume of ~30 mm^3^. At this stage, animals received daily i.v. injections of either saline, SLT-1A^IYSNKLM ^(0.5 mg/kg; Days 8 to 12 and 15 to 19) or an i.p. dose of DTIC (8 mg/kg; Days 8 to 12) or the combination treatment of both SLT-1A^IYSNKLM ^(i.v. dose: 0.5 mg/kg; Days 8 to 12 and 15 to 19) and DTIC (i.p. dose: 8 mg/kg; Days 8 to 12) (7 mice per treatment regimen). Mice were weighed and the dimension of their tumors measured with calipers. Tumor volumes were calculated by assuming a prolate spheroid shape (tumour volume = (large diameter × [short diameter]^2^)/2). Animal survival was monitored over a period of 230 days. Mice were euthanized by exposure to CO_2 _when tumor diameters reached 15 mm, or when ulcerations or other signs of distress such as poor grooming were observed in accordance with the regulatory parameters of the Animal Care Committee at the University Health Network.

### Statistical Analysis

Statistical analyses of tumor growth in mice were performed using one-way analysis of variance (ANOVA). Kaplan-Meier curves were used to summarize the distribution of mouse survival times. We used the Mantel-Cox test to compare the survival curves among treatment groups. All statistical tests were performed using GraphPad Prism 5.0 (GraphPad Software, Inc). *P *values less than 0.05 were considered statistically significant.

## Abbreviations

(RIP): Ribosome-Inactivating Protein; (scRIP): Single Chain Ribosome-Inactivating Protein; (SLT-1): Shiga-Like Toxin 1; (SLT-1A): Shiga-Like Toxin 1 A subunit; (SLT-1A^IYSNKLM^): Shiga-Like Toxin 1 A subunit with an IYSNKLM insertion between residues 245 and 246; (DTIC): Dacarbazine; (PARP): Poly(ADP) Ribose Polymerase; (MIP): Maximum Intensity Projection; (CBCT): Cone-Beam CT; (OSEM): Ordered Subset Expectation Maximization; (PE): *Pseudomonas *exotoxin A; (HMW-MAA): High Molecular Weight Melanoma Associated Antigen; (MCSP): Melanoma Chondroitin Sulfate Proteoglycan; (CXCR4): CXC chemokine receptor; (CEACAM1): Carcinoembryonic-Antigen-Related Cell Adhesion Molecule 1

## Declaration of Competing interests

MCC, SP, XW, DEG and AAW declare that they have no competing interests. RK formerly received a salary from Molecular Templates Inc. LR and JG are founding members of Molecular Templates Inc. Patent applications have been filed for SLT-1A^IYSNKLM ^by the University Health Network (UHN, Toronto, academic entity of JG). UHN recently sold these patent rights to Molecular Templates Inc.

## Authors' contributions

JG conceived the study, participated in its design and coordination, and helped draft the manuscript. MCC designed, carried out and performed the relevant data analysis for the protein purification, cell binding, pharmacokinetics, biodistribution, imaging, densitometry, and serum stability experiments, conducted part of the mouse survival studies, generated the summary figures for the library design and screening, graphed and analyzed the data for the cell cytotoxicity, immunogenicity, and mouse survival studies, and helped draft the manuscript. LR participated in the general supervision of the research group while they collected data for the cell cytotoxicity, immunogenicity, and mouse survival studies. SP designed the preliminary tripeptide library and developed screening approaches, carried out apoptosis experiments as well as performed the relevant data analysis. XW designed and carried out the heptapeptide library design and screening, as well as the initial cell survival studies and identification of the tumour-targeted SLT-1A variant. RK carried out and collected data for the mouse survival and immunogenicity studies as well as performed the protein purification and heptapeptide library screening. DEG participated in the design, coordination, execution, and data analysis for the mouse imaging studies. AAW helped coin the acronym RESCRIPT and assisted in data collection for some of the mouse biodistribution work. All authors read and approved the final manuscript.

## Supplementary Material

Additional file 1 **Figure S1: RESCRIPT: A discovery tool for the Rapid Evolution and Selection of Cancer-Specific Ribosome-Inactivating Protein Toxins**. Diagram outlining the general procedure for the screening and identification of Shiga-like Toxin 1 (SLT-1) A subunit toxin variants with novel cancer-targeting and killing properties. His-tagged toxin variants were individually purified and applied to 96-well plates seeded with cancer cell lines. Cell viability was assessed using a sulforhodamine B (SRB) assay [24]. Click here for file

Additional file 2 Supplementary Text Click here for file

Additional file 3 **Figure S2: The catalytic function of the SLT-1 A subunit was not disrupted by inserting a 7-residue peptide between residues 245 and 246**. (A) The heptapeptide insert did not affect the ribosome-inactivating activity of the purified toxin variants compared to the wt A subunit as measured by the biosynthesis of luciferase (relative light units) in a coupled transcription/translation assay. Legend: wt SLT-1 (open squares), SLT-1A^PDTRPAP ^(open triangles), and a catalytically-inactive SLT-1 variant bearing E167A and R170A mutations within the A subunit (open circles). The peptide insert within the context of the toxin A subunit is exposed and recognized by large proteins such as antibodies. Samples were probed with either an anti-SLT-1 A subunit polyclonal antisera or a PDTRPAP-specific anti-Onc-M27 monoclonal antibody. (B) Western blot analyses of purified A and A_1 _subunits for wt SLT-1 or SLT-1A^PDTRPAP^. (C) ELISA experiments comparing the immunoreactivity of purified AB_5_, A and A_1 _subunits to the *Onc*-M27 mAb for wt SLT-1 and SLT-1A^PDTRPAP ^toxins. Click here for file

Additional file 4 **Figure S3: Serum stability of SLT-1A^IYSNKLM^**. Western blot analysis demonstrating the serum stability of SLT-1A^IYSNKLM ^at 37°C over a period of 24 h. Click here for file

Additional file 5 **Figure S4: Purified SLT-1A^IYSNKLM^**. Coomassie-stained, SDS-PAGE gel showing Lane 1) purified SLT-1A^IYSNKLM ^before furin digestion; Lane 2) purified SLT-1A^IYSNKLM ^after furin treatment; 3) purified wt SLT-1A before furin digestion; Lane 4) purified wt SLT-1A after furin treatment. Click here for file
