# Supplementary figures and images for: An evolved ribosome-inactivating protein targets and kills human melanoma cells in vitro and in vivo
Source: Mol Cancer. 2010 Feb 3;9:28. doi: 10.1186/1476-4598-9-28 (PMC2828990; doi:10.1186/1476-4598-9-28)

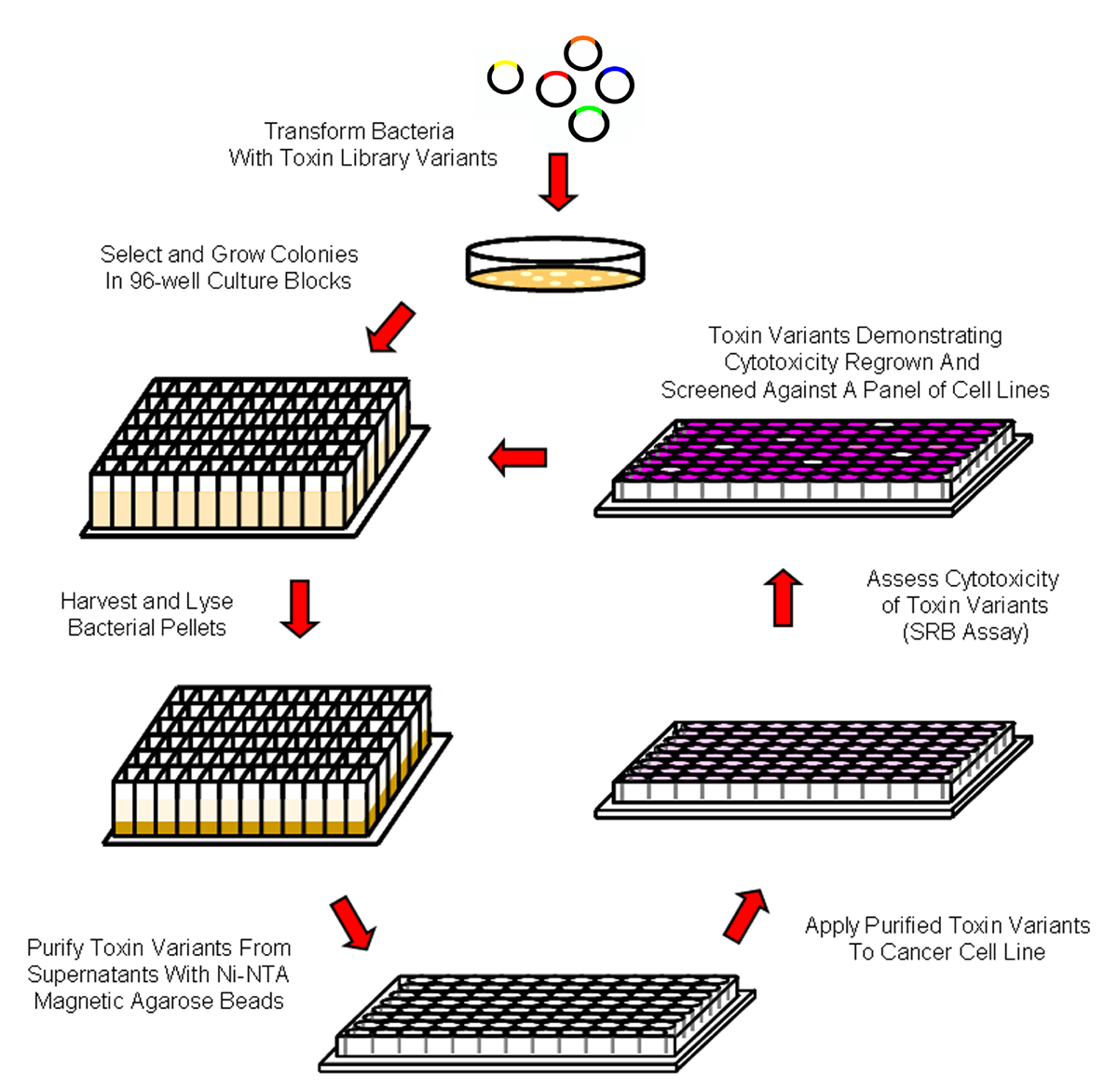

Supplement: Additional file 1 — Figure S1: RESCRIPT: A discovery tool for the Rapid Evolution and Selection of Cancer-Specific Ribosome-Inactivating Protein Toxins. Diagram outlining the general procedure for the screening and identification of Shiga-like Toxin 1 (SLT-1) A subunit toxin variants with novel cancer-targeting and killing properties. His-tagged toxin variants were individually purified and applied to 96-well plates seeded with cancer cell lines. Cell viability was assessed using a sulforhodamine B (SRB) assay [24]. [file 1476-4598-9-28-S1.TIFF]

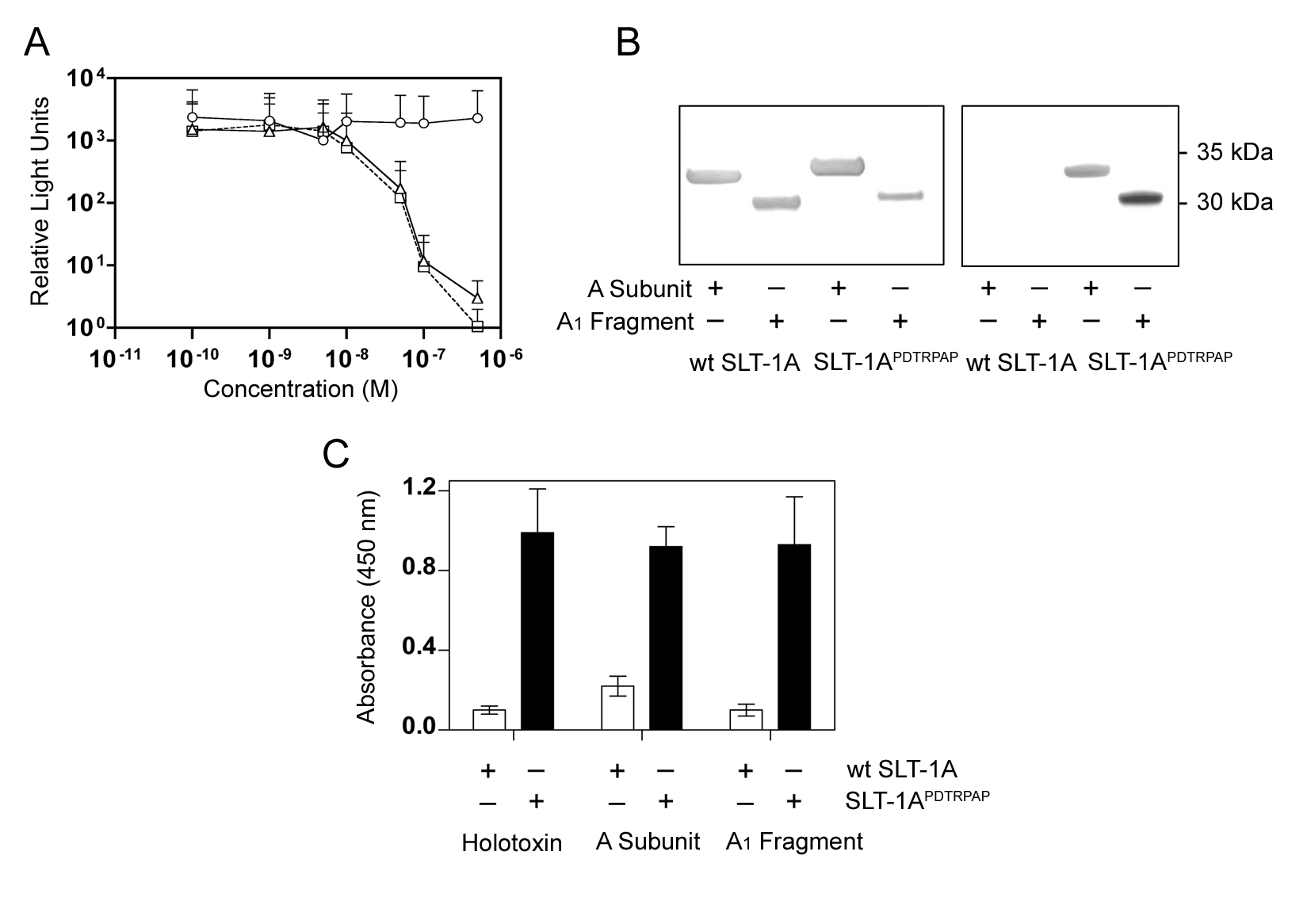

Supplement: Additional file 3 — Figure S2: The catalytic function of the SLT-1 A subunit was not disrupted by inserting a 7-residue peptide between residues 245 and 246. (A) The heptapeptide insert did not affect the ribosome-inactivating activity of the purified toxin variants compared to the wt A subunit as measured by the biosynthesis of luciferase (relative light units) in a coupled transcription/translation assay. Legend: wt SLT-1 (open squares), SLT-1APDTRPAP (open triangles), and a catalytically-inactive SLT-1 variant bearing E167A and R170A mutations within the A subunit (open circles). The peptide insert within the context of the toxin A subunit is exposed and recognized by large proteins such as antibodies. Samples were probed with either an anti-SLT-1 A subunit polyclonal antisera or a PDTRPAP-specific anti-Onc-M27 monoclonal antibody. (B) Western blot analyses of purified A and A1 subunits for wt SLT-1 or SLT-1APDTRPAP. (C) ELISA experiments comparing the immunoreactivity of purified AB5, A and A1 subunits to the Onc-M27 mAb for wt SLT-1 and SLT-1APDTRPAP toxins. [file 1476-4598-9-28-S3.TIFF]

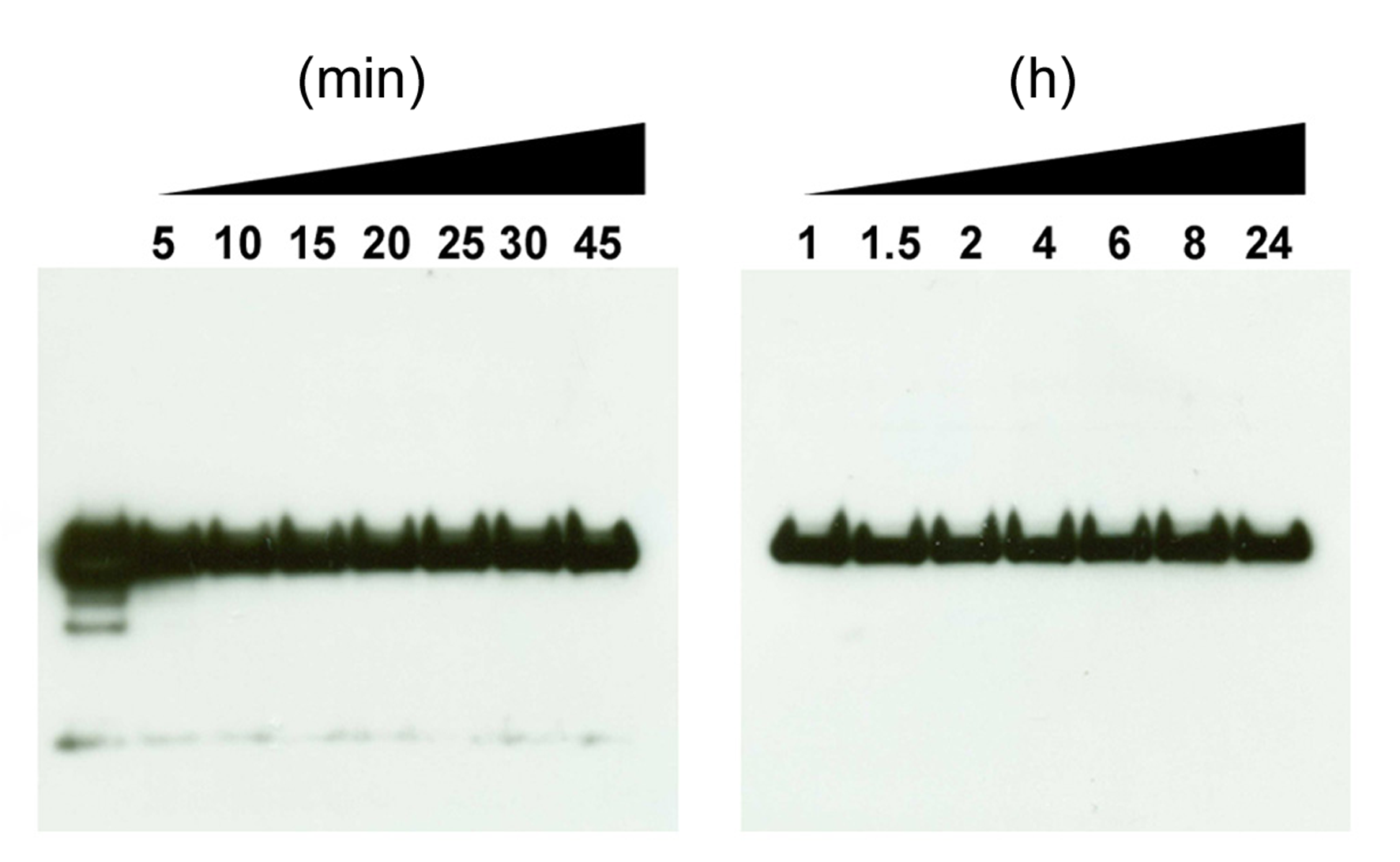

Supplement: Additional file 4 — Figure S3: Serum stability of SLT-1AIYSNKLM. Western blot analysis demonstrating the serum stability of SLT-1AIYSNKLM at 37°C over a period of 24 h. [file 1476-4598-9-28-S4.TIFF]

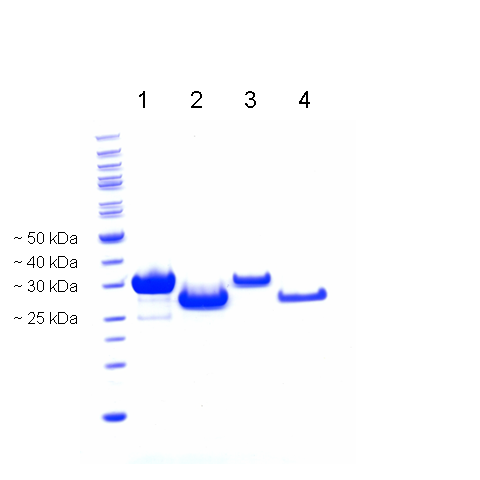

Supplement: Additional file 5 — Figure S4: Purified SLT-1AIYSNKLM. Coomassie-stained, SDS-PAGE gel showing Lane 1) purified SLT-1AIYSNKLM before furin digestion; Lane 2) purified SLT-1AIYSNKLM after furin treatment; 3) purified wt SLT-1A before furin digestion; Lane 4) purified wt SLT-1A after furin treatment. [file 1476-4598-9-28-S5.TIFF]
