# Supplementary material for: An evolved ribosome-inactivating protein targets and kills human melanoma cells in vitro and in vivo
Source: Mol Cancer. 2010 Feb 3;9:28. doi: 10.1186/1476-4598-9-28 (PMC2828990; doi:10.1186/1476-4598-9-28)
Supplement: Additional file 2 — Supplementary Text [file 1476-4598-9-28-S2.DOC]

**ADDITIONAL FILE 1**

**Supplementary Text:**

**Library Design and Validation.** A PCR-based approach was employed to experimentally validate the site of insertion by introducing the 7-amino acid long human mucin (MUC-1) peptide PDTRPAP between residues 245 and 246. This site had to be solvent-exposed (to act as a ligand), while not affecting regions of the A subunit involved in catalysis (N-glycosidase activity), ribosome-binding or ER retro-translocation (see Additional File 1: Figure S1) [1-5]. Surface representation models of the SLT-1 A subunit were derived from the crystal structure of Shiga toxin and indicate that the 7-residue long insertion location is positioned away from the catalytic site of the A subunit (see Additional File 1: Figure S1B) [6-9]. Briefly, two flanking primers E and F containing the restriction sites HindIII and PstI were annealed to the 5’- and 3’-end of the SLT‑1 operon and two mutagenic (overlapping) primers containing the inserted nucleotide sequence were used to build the construct. The 5’-end of the A subunit was amplified with primers E and G, and the 3’-end with primers F and H. The resulting overlapping fragments were annealed at an equimolar ratio followed by amplification of the full cassette with primers E and F. The final PCR product was digested with the restriction enzymes *PstI* and *HindIII* (Invitrogen) and cloned into an SLT-1 constitutive expression vector (pECHE9A). The construction was confirmed by diagnostic restriction analysis with *NsiI*, which indicated the loss of this unique site (ATGCAT) compared to wild-type, and subsequent sequence analysis of the entire cassette region. Primer E: GTT ACT GTG ACA GCT GAA GCT TTA CGT TTT CG; Primer F: GAG AAG AAG AGA CTG CAG ATT CCA TCT GTT G; Primer G: GCT GGT CGC GTG TCT GGA TGA TGA TGA CAA TTC AGT AT; Primer H: GAC ACG CGA CCA GCT CCA GCA TCG CGA GTT GCC AGA ATG.

The catalytic activity of the resulting scRIP variant, SLT-1A^PDTRPAP^, was identical to that of the wild type A subunit, as measured by the biosynthesis of luciferase (RLU/mg protein) in an *in vitro* coupled transcription/translation reticulocyte lysate assay (see Additional File 3: Figure S2A). This result demonstrates that the heptapeptide insertion did not alter the proper folding of residues near the catalytic site of the molecule.

The exposure of the peptide insert within the context of the mutant A subunit and AB_5_ holotoxin was subsequently evaluated by measuring the heptapeptide reactivity towards a monoclonal antibody. Specifically, western blot analyses with the *Onc*-M27 (IgG1) antibody which recognizes the tripeptide epitope TRP in the context of the MUC-1 tandem repeat sequence TRPAP [10] confirmed the accessibility of the inserted peptide to large protein ligands such as antibodies (see Additional File 3: Figure S2B). The presence of the A subunit itself was verified by Western blot analyses using a rabbit polyclonal antiserum recognizing the SLT-1 A subunit (see Additional File 3: Figure S2B). The exposure of the heptapeptide within SLT-1A^PDTRPAP^ was also confirmed by ELISA (see Additional File 3: Figure S2C).

Generation of a Catalytically-Inactive SLT-1A^IYSNKLM^. Tyrosine 77 within the A subunit of wt SLT-1 was mutated to a serine (Y77S) within the scaffold of the SLT-1A^IYSNKLM^ variant using the quick change site-directed mutagenesis kit (Stratagene). The resulting construct was catalytically inactive as measured using a coupled transcription/translation assay (TNT assay; Promega).

Radioligand Binding Assays. The equilibrium dissociation constant of ^125^I-SLT-1A^IYSNKLM^ was first derived *in vitro* from a series of competition binding curves. Specifically, human melanoma 518-A2 cells were incubated at 4°C with ^125^I-SLT-1A^IYSNKLM^ ranging in concentration from 1.2 x 10^-9^ M to 1.2 x 10^-6^ M as well as an excess of unlabeled SLT-1A^IYSNKLM^ ranging from 4.7 x 10^‑9^ M to 4.7 x 10^-7^ M. Each competitive binding curve was used to determine the specific binding value (SB) where $SB=Nonspecific+\left[(Total-Nonspecific)/(1+10^{\log[D]-\log[IC50]})\right]$ and D represents the saturating concentration of unlabeled SLT-1A^IYSNKLM^. The analysis provides an equilibrium dissociation constant (K_a_) for ^125^I-SLT-1A^IYSNKLM^ of (1.4 ± 0.2) x 10^-7^ M and the presence of (1.4 ± 0.1) x 10^5^ binding sites/cell on 518-A2 human melanoma cells. The inhibitory concentration of unlabeled SLT-1A^IYSNKLM^ leading to 50% displacement of bound ^125^I‑SLT‑1A^IYSNKLM^ (IC_50_) was calculated to be (2.4 ± 0.3) x 10^-8^ M. The dissociation constant (K_d_) of unlabeled SLT-1A^IYSNKLM^ was then calculated to be (1.8 ± 0.3) x 10^-8^ M using the equation K_d_ = IC_50_ / [1 + (a / K_a_)] where the term a is the concentration of ^125^I-SLT-1A^IYSNKLM^. Displacement curves were analyzed using GraphPad Prism 5.0 (GraphPad Software, Inc).

**Imaging Studies**. Tumor-bearing SCID mice received an i.v. dose (900 µCi) of either ^125^I‑SLT‑1A^IYSNKLM^ or ^125^I-SLT-1A as measured in a Capintec CRC15 dose calibrator. A cone-beam CT (CBCT) followed by helical SPECT was acquired using a dual-modality small animal NanoSPECT/CT (Bioscan). The SPECT scanner was equipped with four standard tungsten apertures (9-pinholes, 1.4 mm diameter) that projected the gamma-rays to their respective sodium-iodide detectors (230 × 215 mm), each connected to 33 PMTs. Helical SPECT scans were acquired using 24 projections with a minimum of 50,000 cps/detector. The mice were kept conscious for 10 min post-injection prior to being anesthetized for scanning using 1 L/min oxygen flow containing 5% isofluorane for induction and 2% isofluorane for maintenance. The CBCTs were acquired ~15-25 min post-injection (180 projections, 1 s/projection, 45 kVp, 177 μA) for anatomical reference of the Iodine-125 radiopharmaceutical accumulation. SPECT scans were acquired 30-60 min post-injection. Images were reconstructed using an ordered subset expectation maximization (OSEM) algorithm (9 iterations). SPECT and CT images were co-registered and visualized using InvivoScope (Version 1.37, Bioscan) and a consistent color scale was applied to all SPECT images. All animal protocols were reviewed and approved by the University Health Network’s Animal Care Committee.

**Human Serum Stability.** The chemical stability of SLT-1A^IYSNKLM^ was assessed by incubating 535 µg of SLT-1A^IYSNKLM^ in 9 mL of human serum (Sigma-Aldrich) at 37°C. Aliquots of 300 μL were removed at multiple time points over a period of 24 h then incubated for 1 h at room temperature with 70 μL of Ni-NTA Magnetic Agarose beads (Qiagen) pre-equilibrated with phosphate Buffer A (50 mM phosphate buffer, pH 8, 300 mM NaCl, and 10 mM imidazole, supplemented with 0.1% v/v Tween‑20). Protein-bound beads were washed three times in phosphate Buffer B (same as Buffer A but with 20 mM imidazole, supplemented with 0.1% v/v Tween-20). Protein was then eluted with 40 μL of phosphate Buffer C (same as Buffer A but with 250 mM imidazole, supplemented with 0.1% v/v Tween-20). Samples were analyzed by Western blot using a rabbit polyclonal anti-serum recognizing the SLT-1 A subunit at 1:1,000 and a 1:3,000 dilution of goat anti-mouse IgG-peroxidase conjugate (Sigma-Aldrich).

**References:**

1. Bray MR, Bisland S, Perampalam S, Lim WM, Gariepy J: **Probing the surface of eukaryotic cells using combinatorial toxin libraries**. *Curr Biol* 2001, **11**(9):697-701.

2. Garred O, Dubinina E, Polesskaya A, Olsnes S, Kozlov J, Sandvig K: **Role of the disulfide bond in Shiga toxin A-chain for toxin entry into cells**. *J Biol Chem* 1997, **272**(17):11414-11419.

3. LaPointe P, Wei X, Gariepy J: **A role for the protease-sensitive loop region of Shiga-like toxin 1 in the retrotranslocation of its A1 domain from the endoplasmic reticulum lumen**. *J Biol Chem* 2005, **280**(24):23310-23318.

4. McCluskey AJ, Poon GM, Bolewska-Pedyczak E, Srikumar T, Jeram SM, Raught B, Gariepy J: **The catalytic subunit of shiga-like toxin 1 interacts with ribosomal stalk proteins and is inhibited by their conserved C-terminal domain**. *J Mol Biol* 2008, **378**(2):375-386.

5. Sandvig K, van Deurs B: **Transport of protein toxins into cells: pathways used by ricin, cholera toxin and Shiga toxin**. *FEBS Lett* 2002, **529**(1):49-53.

6. Fraser ME, Chernaia MM, Kozlov YV, James MN: **Crystal structure of the holotoxin from Shigella dysenteriae at 2.5 A resolution**. *Nat Struct Biol* 1994, **1**(1):59-64.

7. Deresiewicz RL, Calderwood SB, Robertus JD, Collier RJ: **Mutations affecting the activity of the Shiga-like toxin I A-chain**. *Biochemistry* 1992, **31**(12):3272-3280.

8. Deresiewicz RL, Austin PR, Hovde CJ: **The role of tyrosine-114 in the enzymatic activity of the Shiga-like toxin I A-chain**. *Mol Gen Genet* 1993, **241**(3-4):467-473.

9. Hovde CJ, Calderwood SB, Mekalanos JJ, Collier RJ: **Evidence that glutamic acid 167 is an active-site residue of Shiga-like toxin I**. *Proc Natl Acad Sci U S A* 1988, **85**(8):2568-2572.

10. Linsley PS, Brown JP, Magnani JL, Horn D: **Monoclonal antibodies reactive with mucin glycoproteins found in sera from breast cancer patients**. *Cancer Res* 1988, **48**(8):2138-2148.
